# FGFRL1 is a neglected putative actor of the FGF signalling pathway present in all major metazoan phyla

**DOI:** 10.1186/1471-2148-9-226

**Published:** 2009-09-09

**Authors:** Stephanie Bertrand, Ildiko Somorjai, Jordi Garcia-Fernandez, Thomas Lamonerie, Hector Escriva

**Affiliations:** 1 Departament de Genètica, Facultat de Biologia, Universitat de Barcelona, Av. Diagonal 645, edifici annex, 1a planta, 08028 Barcelona, España; 2 CNRS UMR 7628, UPMC Univ Paris 06, Observatoire océanographique, F-66651 Banyuls-sur-Mer, France; 3 Université de Nice-Sophia Antipolis, Institute of Developmental Biology and Cancer, CNRS UMR 6543, Faculté des Sciences-Parc Valrose, 06108 Nice Cedex 2, France

## Abstract

**Background:**

Fibroblast Growth Factors (FGF) and their receptors are well known for having major implications in cell signalling controlling embryonic development. Recently, a gene coding for a protein closely related to FGFRs (Fibroblast Growth Factor Receptors) called FGFR5 or FGFR-like 1 (FGFRL1), has been described in vertebrates. An orthologous gene was also found in the cephalochordate amphioxus, but no orthologous genes were found by the authors in other non-vertebrate species, even if a FGFRL1 gene was identified in the sea urchin genome, as well as a closely related gene, named *nou-darake*, in the planarian *Dugesia japonica*. These intriguing data of a deuterostome-specific gene that might be implicated in FGF signalling prompted us to search for putative FGFRL1 orthologues in the completely sequenced genomes of metazoans.

**Results:**

We found FGFRL1 genes in the cnidarian *Nematostella vectensis *as well as in many bilaterian species. Our analysis also shows that FGFRL1 orthologous genes are linked in the genome with other members of the FGF signalling pathway from cnidarians to bilaterians (distance < 10 Mb). To better understand the implication of FGFRL1 genes in chordate embryonic development, we have analyzed expression patterns of the amphioxus and the mouse genes by whole mount *in situ *hybridization. We show that some homologous expression territories can be defined, and we propose that FGFRL1 and FGF8/17/18 were already co-expressed in the pharyngeal endoderm in the ancestor of chordates.

**Conclusion:**

Our work sheds light on the existence of a putative FGF signalling pathway actor present in the ancestor of probably all metazoans, the function of which has received little attention until now.

## Background

Interaction between many different signalling pathways is necessary to form a metazoan starting from a single egg cell. Fibroblast Growth Factor (FGF) signalling represents one such developmental pathway. FGFs are small proteins that act by binding to their transmembrane receptors, FGFRs. The latter are characterised by three Immunoglobulin-like (Ig-like) extracellular domains, which are implicated in ligand and heparan sulphate binding, as well as an intracellular tyrosine kinase domain responsible for signal transduction. In mammals, the analysis of several completely sequenced genomes shows the presence of 22 FGF and 4 FGFR genes [1]. Interestingly, FGFs and FGFRs arose early in metazoan evolution, since conserved genes for the two families are present in the genomes of diploblastic animals like the sea anemone *Nematostella vectensis *[2].

Besides the 4 classical FGFRs known in mammals, a fifth evolutionarily related protein, called FGFR5 or FGFR-like 1 (FGFRL1), has recently been described in vertebrates [3-8]. FGFRL1 displays the same structural organization as FGFRs, with the exception of the cytoplasmic tyrosine kinase domain. Indeed, this receptor has a signal peptide followed by three Ig-like loops and a transmembrane domain. The cytoplasmic part of the protein is not related to tyrosine kinases and does not show any known structural feature that could help to understand its function. Using GFP-fused receptors, it was shown that FGFRL1 is localised to the membrane, and FRET assays showed that FGFRL1 forms homodimers through interactions implicating both the extracellular and intracellular domains [9]. However, using the same technique, no interaction between FGFRL1 and FGFRs could be detected [9]. Moreover, a variety of assays show that FGFRL1 is able to bind at least FGF2, but with lower affinity than FGFRs, and that it also seems to interact with heparan sulphate [4,6]. In light of all these data, it was proposed that FGFRL1 might act as a "decoy" receptor for FGFR by trapping FGFs.

Gene inactivation experiments performed in mouse and zebrafish have shed light on the possible *in vivo *function of FGFRL1 during development [5,10-12]. In *Danio rerio*, two copies of FGFRL1 have been identified, *fgfrl1a *and *fgfrl1b*, which probably arose after the genome duplication that took place early in the evolution of actinopterygian fish lineage [5]. Injection of morpholinos targeting one or both duplicates leads to defects in cartilage formation of the pharyngeal arches [10]. No other defects were described, a surprising result given that *fgfrl1a *and *fgfrl1b *mRNAs are detected in other organs such as pectoral fin buds and lens, as well as in the midbrain-hindbrain boundary. However, *fgfrl1a*-morpholino injected embryos have a phenotype resembling that of FGF3 mutants that is not consistent with a putative dominant negative activity of FGFRL1 [10]. On the other hand, the first knock-out mutant mice described for the FGFRL1 gene show a very mild phenotype in homozygous embryos [11]. Indeed, newborns seem normal but die one hour after birth due to respiratory failure associated with an abnormally-formed diaphragm. However, a recent publication, describing the phenotype of other knock-out mutant mice for the FGFRL1 gene, shows that disrupting this gene also leads to craniofacial defects [12]. This suggests that the implication of FGFRL1 in pharyngeal development may be conserved in vertebrates.

An orthologue of FGFRL1 was isolated in the cephalochordate amphioxus *Branchiostoma floridae *[13], but searches for orthologous genes in the completely sequenced genomes of other non vertebrates were unsuccessful. This was surprising given the description of the *nou-darake *gene in the planarian *Dugesia japonica*, which clearly shows a structure related to FGFRL1 [14]. It was therefore proposed that FGFRL1 was specific to the chordate lineage. However, an FGFRL1 orthologue was later identified in the genome of the sea urchin [15]. In order to gain insight into the origin and evolution of the FGFRL1 gene family, we performed an exhaustive Blast analysis. We found at least one FGFRL1 orthologue in many invertebrate species, including the nematode *Caenorhabditis elegans*, the sea squirt *Ciona intestinalis*, and the crustacean *Daphnia pulex*. We also identified a clear FGFRL1 orthologue in the genome of *Nematostella vectensis*. To further analyse the role of this gene, which has been conserved from diploblastic animals to mammals, and to better understand its ancestral function in chordates, we characterised mRNA expression during amphioxus embryonic development, as well as in early embryonic stages in mouse. Our data highlight two embryonic regions that might correspond to FGFRL1 expression territories in the ancestor of chordates, namely the pharyngeal endoderm and the anterior neural tissues. Finally, our work sheds light on a possible new actor of the FGF signalling pathway that has been poorly studied until now, particularly outside vertebrates.

## Results

### Nou-darake from Dugesia japonica is a real FGFRL1 orthologue

**Figure 1:**
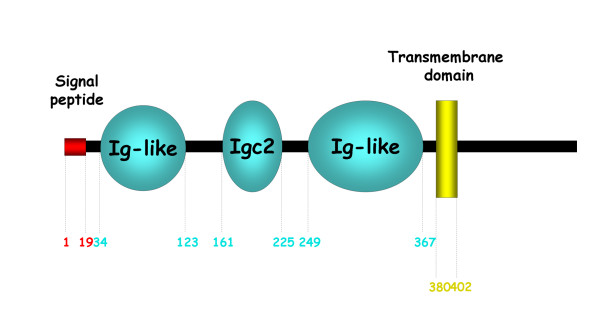
**Domain organization of the planarian *nou-darake *protein**. Domain organization follows that proposed by the SMART online software. Positions at the beginning and end of each domain in the protein sequence are indicated. Ig: Immunoglobulin.

**Figure 2:**
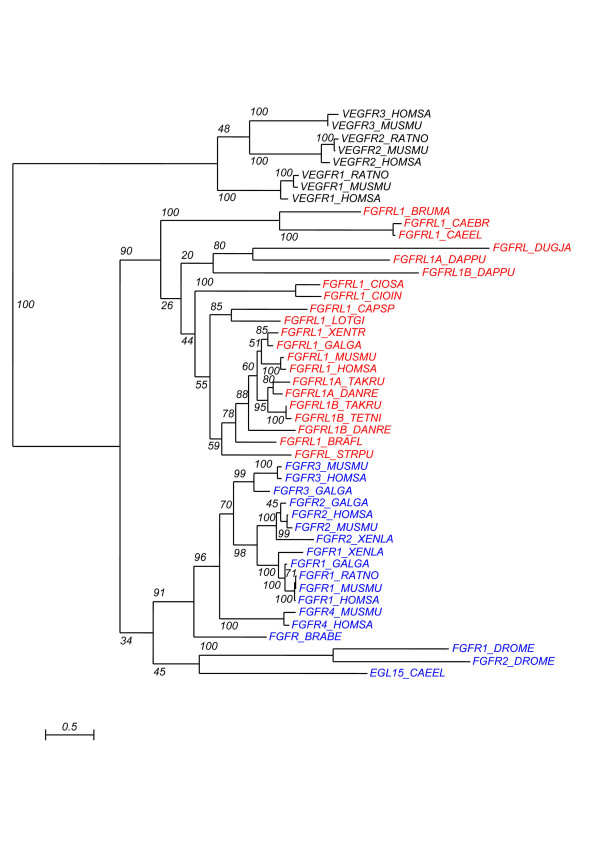
**Phylogenetic analysis of the FGFRL1 putative orthologues**. Phylogenetic tree estimated under the JTT+I+G model (RAxML with rapid bootstrap analysis with 100 bootstrapping runs). VEGFR sequences from vertebrates were used as the outgroup. FGFR sequences are indicated in blue and FGFRL1 sequences in red. The species abbreviations are as follows: *BRABE: Branchiostoma belcheri, BRAFL: Branchiostoma floridae, BRUMA: Brugia malayi, CAEBR: Caenorhabditis briggsae, CAEEL: Caenorhabditis elegans, CAPSP: Capitella sp.I, CIOIN: Ciona intestinalis, CIOSA: Ciona savignyi, DANRE: Danio rerio, DAPPU: Daphnia pulex, DROME: Drosophila melanogaster, DUGJA: Dugesia japonica, GALGA: Gallus gallus, HOMSA: Homo sapiens, LOTGI: Lottia gigantea, MUSMU: Mus musculus, RATNO: Rattus norvegicus, STRPU: Strongylocentrotus purpuratus, TAKRU: Takifugu rubripes, TETNI: Tetraodon nigroviridis, XENLA: Xenopus laevis, XENTR: Xenopus tropicalis*.

We used online SMART software  to analyse the domain organization of the *nou-darake *protein from the planarian *Dugesia japonica *and found that contrary to previous descriptions, the protein possesses not two but three Ig loops [16,17]. The complete structure is composed of a signal peptide followed by 3 Ig loops and a transmembrane domain, similarly to FGFRL1 (see Figure 1). A comparison by BlastP of the amino acid sequence of *nou-darake *with the non-redundant CDS database from Genbank yielded vertebrate and amphioxus FGFRL1 sequences as best reciprocal hits. We also performed a phylogenetic reconstruction for FGFR and FGFRL1 families. The topology of the tree we obtained clearly shows that *nou-darake *is more closely related to the FGFRL1 family than to FGFRs (Figure 2). The data therefore clearly identify *nou-darake *as a true orthologue of FGFRL1.

These findings clearly establish that FGFRL1 is not only present in chordates and echinoderms, but also in lophotrochozoans. Although the possibility of a high rate of gene loss in many animals cannot be excluded, it was surprising that no orthologue was found in any completely sequenced genome except for those of vertebrates and of the sea urchin. We therefore undertook a more detailed analysis of the other available genomes.

### FGFRL1 orthologues are present in many metazoans

We searched for genes that might be potential orthologues of FGFRL1 in several completely sequenced genomes from invertebrate species (see Table 1 for details), as well as in the Genbank database using a classical Blast approach. As shown in Table 1, we found putative orthologues in almost all the species we have analysed, from a diploblastic animal, the sea anemone *Nematostella vectensis*, to echinoderms (*Strongylocentrotus purpuratus*) and tunicates (*Ciona savignyi *and *Ciona intestinalis*). Some of the sequences we obtained are complete CDS, whereas others are incomplete, probably as a result of prediction/assembly mistakes or incomplete sequencing. We also looked at the genome sequences of vertebrate species for which FGFRL1 was described to ensure there were no additional paralogues. As previously described, we only found one gene in human, mouse, chicken and *Xenopus*, and two genes in zebrafish as well as in *Tetraodon *and *Takifugu *(Table 2). In invertebrates, we found a single putative FGFRL1 orthologue for each species, except in *Daphnia pulex *which has at least 3 genes, named A, B and C. Gene predictions are not complete for the three genes. They are all placed close together in the same contig and may correspond to specific tandem duplications in this species. We also looked for putative FGFRL1 orthologues in the genome of the choanoflagellate *Monosiga brevicolis*. Previous reports indicate that *M. brevicolis *has few Ig domain-containing proteins [18], and our analysis suggests that none of these correspond to FGFRL1 orthologues.

**Table 1:** Species for which we searched for FGFRL1 putative orthologues.

**Species**	**Group**	**Web site**	**Number of orthologues**
*Aedes aegypti*	Insect		1
*Anopheles gambiae*	Insect		1
*Branchiostoma floridae*	Cephalochordate		1
*Brugia malayi*	Nematode		1
*Caenorhabditis elegans*	Nematode		1
*Caenorhabditis briggsae*	Nematode		1
*Capitella sp. I*	Annelid		1
*Ciona intestinalis*	Tunicate		1
*Ciona savigny*	Tunicate		1
*Danio rerio*	Vertebrate		2
*Daphnia pulex*	Crustacean		3
*Drosophila melanogaster*	Insect		1
*Drosophila pseudoobscura*	Insect		1
*Dugesia japonica*	Planarian		1
*Helobdella robusta*	Annelid		0
*Homo sapiens*	Vertebrate		1
*Lottia gigantea*	Mollusc		1
*Monosiga brevicollis*	Choanoflagellate		0
*Mus musculus*	Vertebrate		1
*Nematostella vectensis*	Anthozoan		1
*Strongylocentrotus purpuratus*	Echinoderm		1
*Tetraodon nigroviridis*	Vertebrate		2
*Takifugu rubripes*	Vertebrate		2
*Tribolium castaneum*	Insect		1
*Xenopus tropicalis*	Vertebrate		1

For each species the name of the group, the website used in this study as well as the number of putative orthologues are given.

**Table 2:** Putative FGFRL1 orthologues.

**Species**	**Name**	**Accesion number**	**Domain organization**	**Best reciprocal Blast with vertebrates**
*Aedes aegypti*	FGFRL1_AEDAE	XP_001663291.1	SP (1-23), Ig (62-174), Ig (192-301), TM (317-339)	T. rubripes FGFRL1a (CAH03726)
*Anopheles gambiae*	FGFRL1_ANOGA	XP_313121.4	SP (1-30), Ig (42-154), Ig (178-267), TM (298-320)	T. rubripes FGFRL1a (CAH03726)
*Branchiostoma floridae*	FGFRL1_BRAFL	CAI61931.1	SP (1-17), Ig (35-99), Ig (145-211), Ig (236-340), TM (367-389)	X. laevis FGFRL1 (AAI69825)
*Brugia malayi*	FGFRL1_BRUMA	EDP33612.1	Ig (2-50), Ig (103-166), Ig (210-301), TM (325-347)	D. rerio FGFRL1b (CAH03196)
*Caenorhabditis elegans*	FGFRL1_CAEEL	Y102A11A.8	SP (1-19), Ig (47-114), Ig (164-225), Ig (267-374), TM (388-410)	R. norevegicus FGFRL1 (NP_954545)
*Caenorhabditis briggsae*	FGFRL1_CAEBR	XP_001675941.1	SP (1-18), Ig (47-115), Ig (165-226), Ig (268-375), TM (389-411)	R. norevegicus FGFRL1 (NP_954545)
*Capitella sp. I*	FGFRL1_CAPSP	**Capca1:170033**	SP (1-21), Ig (39-103), Ig (166-234), Ig (265-352), TM (386-408)	D. rerio FGFRL1a (NP_956670)
*Ciona intestinalis*	FGFRL1_CIOIN	**ENSCINP00000023002**	Ig (12-77), Ig (138-202), Ig (227-336)	H. sapiens FGFRL1 (AAK26742)
*Ciona savigny*	FGFRL1_CIOSA	**SINCSAVP00000006520**	Ig (15-80), Ig (137-201), Ig (226-335)	R. norevegicus FGFRL1 (NP_954545)
*Danio rerio*	FGFRL1A_DANRE	NP_956670.1	SP (1-19), Ig (37-101), Ig (157-222), Ig (253-339), TM (370-392)	
	FGFRL1B_DANRE	NP_001012263.1	SP (1-22), Ig (39-103), Ig (164-227), Ig (258-355), TM (375-397)	
*Daphnia pulex*	FGFRL1A_DAPPU	**Dappu1:109409**	SP (1-23), Ig (40-106), Ig (153-239), Ig (272-379), TM (419-441)	G. gallus FGFRL1 (NP_989787)
	FGFRL1B_DAPPU	**Dappu1:109413**	SP (1-27), Ig (38-121), Ig (164-236), Ig (259-373), TM (386-408)	D. rerio FGFRL1b (AAI62498)
	FGFRL1C_DAPPU	**Dappu1:57798**	Ig (18-82), Ig (150-211), Ig (236-343)	X. laevis FGFRL1 (AAI69825)
*Dugesia japonica*	FGFRL1_DUGJA	BAC20953.1	SP (1-19), Ig (34-123), Ig (161-225), Ig (249-367), TM (380-402)	T. rubripes FGFRL1a (CAH03726)
*Drosophila melanogaster*	FGFRL1_DROME	NP_732672.1	SP (1-29), Ig (38-137), Ig (159-275), TM (306-328)	D. rerio FGFRL1b (CAM60089)
*Drosophila pseudoobscura*	FGFRL1_DROPS	XP_001358156.1	SP (1-17), Ig (26-125), Ig (147-263), TM (293-315)	H. sapiens FGFRL1 (AAK26742)
*Gallus gallus*	FGFRL1_GALGA	CAD59380.1	SP (1-18), Ig (36-100), Ig (157-222), Ig (253-339), TM (370-392)	
*Homo sapiens*	FGFRL1_HOMSA	NP_068742.2	SP (1-24), Ig (42-106), Ig (163-228), Ig (259-356), TM (377-399)	
*Lottia gigantea*	FGFRL1_LOTGI	**Lotgi1:167118**	SP (1-31), Ig (48-113), Ig (167-231), Ig (262-349), TM (381-403)	X. tropicalis FGFRL1 (NP_001011189)
*Mus musculus*	FGFRL1_MUSMU	NP_473412	SP (1-20), Ig (38-102), Ig (159-224), Ig (255-341), TM (372-394)	
*Nematostella vectensis*	FGFRL1_NEMVE	XP_001635234.1	SP (1-28), Ig (44-122), Ig (175-240), Ig (277-388), TM (417-439)	T. nigroviridis FGFRL1b (CAG10558)
*Strongylocentrotus purpuratus*	FGFRL1_STRCE	XP_783945.2	Ig (69-133), Ig (192-255), Ig (280-382), TM (415-437)	H. sapiens FGFRL1 (AAK26742)
*Takifugu rubripes*	FGFRL1A_TAKRU	CAH03726	Ig (15-79), Ig (135-204), Ig (235-321), TM (352-374)	
	FGFRL1B_TAKRU	CAH03727	Ig (20-84), Ig (143-208), Ig (239-325), TM (356-378)	
*Tetraodon nigroviridis*	FGFRL1A_TETNI	CAF96974	Ig (18-84), Ig (115-200), TM (231-253)	
	FGFRL1B_TETNI	CAG10558	Ig (22-86), Ig (145-210), Ig (241-327), TM (358-380)	
*Tribolium castaneum*	FGFRL1_TRICA	XP_967276.1	SP (1-17), Ig (37-125), Ig (142-241), TM (253-275)	T. rubripes FGFRL1a (CAH03726)
*Xenopus tropicalis*	FGFRL1_XENTR	NP_001011189.1	SP (1-21), Ig (36-100), Ig (157-222), Ig (253-391), TM (369-391)	

The accession number in Genbank (black) or in the genomic databases (bold), the domain organization of the protein as proposed by SMART online software, and the best reciprocal blast on vertebrates sequences are given for each gene. The names correspond to the ones used in this study.

The domain organization of the different putative orthologues is given in Table 2, according to the SMART online software [16,17]. All the sequences that are full-length possess three Ig loops, except those found in insects. Indeed, for all insects that we analyzed, putative FGFRL1 orthologous proteins only have two Ig loops between their signal peptide and transmembrane domain.

To ensure that we had found real FGFRL1 orthologues, we performed several phylogenetic analyses. Only the extracellular and transmembrane domains can be aligned and were therefore used for phylogenetic reconstructions. We found that the genes that we isolated for all taxa, with the exception of insects and *Nematostella*, can be clearly placed in the tree with the known deuterostome FGFRL1 (see Figure 2). For the insect sequences, some phylogenetic analyses place them with the FGFRL1 group, but with low bootstrap values (see Additional file 1). This may be due to the fact that there are only two Ig domains and that the sequences are very divergent in these species. However, the structure of the protein, combined with the fact that the best reciprocal Blast hits with vertebrates are FGFRL1 sequences, suggest that the insect genes represent real FGFRL1 orthologues. For *Nematostella vectensis*, the putative FGFRL1 orthologue is placed with the FGFRb sequences from the same species in our phylogenetic reconstructions (see Additional files 1 and 2). Both sequences are positioned at the root of FGFR or FGFRL1 group depending on the method used for phylogenetic reconstruction, and always with a low bootstrap value. However, as for the insect sequences, the structure of the protein, and the fact that the best reciprocal Blast hits with vertebrates are FGFRL1 sequences, support the fact that we have found the *bona fide *FGFRL1 orthologue in *Nematostella*. The position of the sequence in the tree reconstructions may indicate that the FGFR and FGFRL1 genes are the result of the duplication of an ancestral gene very early during metazoan evolution, just before the diploblastic/bilaterian divergence, as has previously been proposed for other tyrosine kinase genes [19].

### FGFRL1 gene organization is conserved between cnidarians and vertebrates

**Figure 3:**
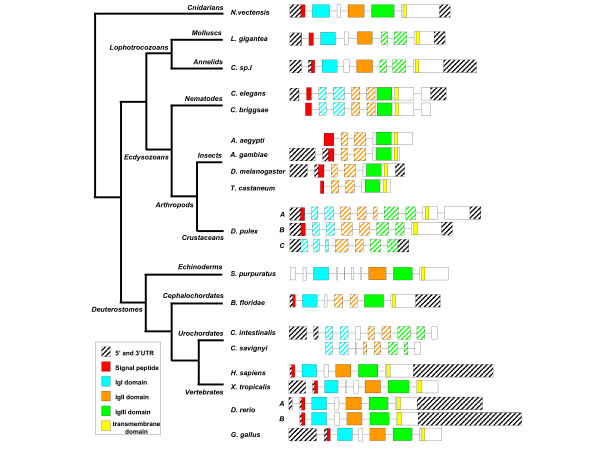
**Schematic representation of the exon/intron organization of the FGFRL1 orthologous genes in different metazoan species**. A schematic phylogenetic tree on the left indicates the evolutionary position of the different species studied. Boxes represent exons. Only exon size is proportional. Colour code is indicated in the figure. The colour of an exon means it contains the full (colour), or partial (hatched) corresponding domain. The exon/intron structures correspond to the data available in the jgi , Genbank , and ensembl  databases.

We have analyzed exon/intron structure for the species for which genomic data are available, the results of which are shown in Figure 3 (*T. rubripes *and *T. nigroviridis *data were omitted for the sake of clarity). Interestingly, in *Nematostella vectensis*, as in human, the gene is encoded by 6 exons. The first exon includes the 5'UTR as well as the signal peptide sequence. Each Ig loop domain is coded by a unique exon, namely exons 2, 4 and 5 for IgI, II and III respectively. Exon 3 codes for a box between IgI and IgII, while the sixth exon codes for the transmembrane as well as the cytoplasmic and 3'UTR sequences. In *Nematostella*, similarly to human, the exon/intron limit ends at phase 1 for exons 1 to 5. Thus, exon/intron structure of FGFRL1 genes is highly conserved between human and *Nematostella*. In the other vertebrate species analysed, the gene organisation is conserved overall, with few differences. Indeed, for *Xenopus*, chick, and the *fgfrl1a *zebrafish duplicate, there is an additional exon coding for part of the 5'UTR. Furthermore, in *Xenopus*, the box between IgI and IgII is encoded not by one, but by two exons. As in human and *Nematostella*, all the exon/intron limits end at phase 1, except for *Xenopus *exon 3. This conservation of genomic structure between sea anemone and vertebrate genes further confirms their putative orthology.

In the deuterostome group, although not all gene predictions are complete, the exon/intron structure is also quite similar to that of vertebrates, except in the urochordates. Indeed, in the sea urchin the only obvious difference is that the box between IgI and IgII is coded by four exons instead of one. In amphioxus, it is the IgII domain that is cut into two exons compared to one in vertebrates and sea urchin [13]. For the two *Ciona *species, the exon/intron data available are incomplete; however, we observed that the gene organization is clearly not conserved with that of the other deuterostomes. Indeed, the three Ig loop coding domains are separated into two exons.

In lophotrochozoans (a mollusc *Lottia gigantea *and an annelid *Capitella species I*), the gene structure is close to that observed for both vertebrates and the sea anemone. In effect, only what corresponds to the first exon is separated into two sections, as well as the IgIII domain coding part, which is split into two exons.

In contrast, the gene structure of all ecdysozoan FGFRL1 orthologues is very different. For *C. elegans *and *C. briggsae*, IgI and IgII domains are each encoded by two exons, whereas the end of IgII, IgIII, the transmembrane domain and part of the cytoplasmic domain are encoded by a unique exon. The last exon codes for the end of the cytoplasmic domain and for the 3'UTR. In *Drosophila*, the first Ig domain is encoded by two exons, while the IgII domain, the transmembrane and cytoplasmic domains as well as the 3'UTR are encoded by a unique exon. In the other insects, for which the gene organization data are incomplete, the exon/intron structure is similar to what is observed in *Drosophila*. Finally, in *Daphnia pulex*, all the Ig domains are encoded by several exons in the three genes found in its genome. The divergence of the genomic structure in several ecdysozoan lineages compared to vertebrates and diploblastic animals has previously been described, and it seems that the FGFRL1 orthologues are no exception [20].

### Genomic linkage of FGFRL1 and genes involved in FGF signalling

**Figure 4:**
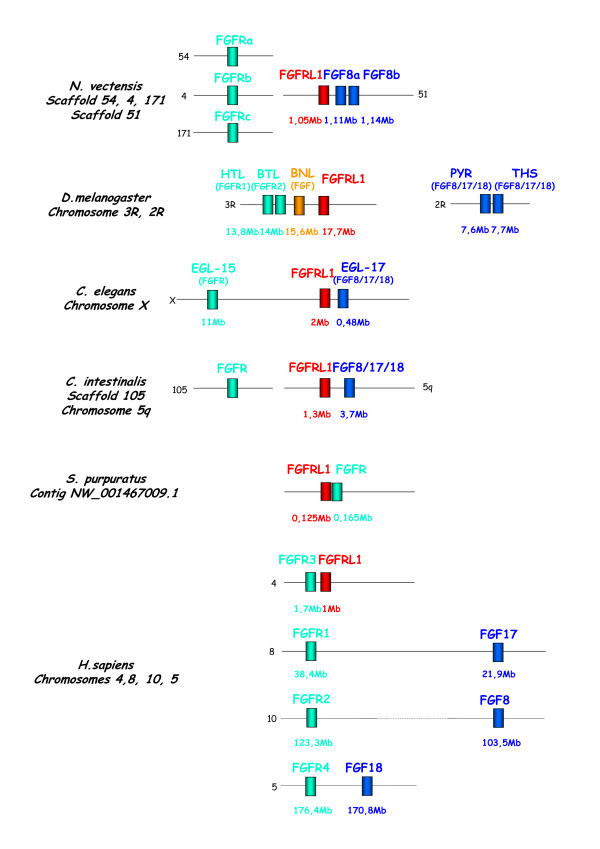
**Genomic position of the FGFRL1, FGF8/17/18 and FGFR orthologues in several metazoan species**. Orthologous genes are schematized by boxes with the same colour. The position on the chromosomes or scaffolds is given according to the jgi , Genbank , and ensembl  databases. Orthology for the *Drosophila *and nematode genes is given in parentheses. HTL: *heartless*, BTL: *breathless*, BNL: *branchless*, THS:*thisbe*, PYR: *pyramus*, EGL: EGg Laying defective.

We have analyzed, when data were available, the genomic position of the FGFRL1 genes and the presence of known FGF signalling pathway genes in their vicinity. Figure 4 summarizes the data for the species for which we find a genomic linkage between FGFRL1 and FGF signalling pathway genes. For the purposes of clarity, only human data are presented for vertebrates. In *Nematostella vectensis*, the FGFRL1 gene is positioned close to the FGF8a and FGF8b genes (defined as <10 Mb), which are orthologous to the vertebrate FGF8/17/18 group. In *Caenorhabditis elegans *and *Ciona intestinalis *(and *Ciona savignyi*, data not shown), FGFRL1 orthologue genes are also placed in close proximity to the FGF8/17/18 orthologues (named egl-17 for the nematode). In contrast, the FGFRL1 orthologue is close to the FGFR orthologues (htl and btl) in *Drosophila*, to the FGFR1 gene in the sea urchin genome, and to the FGFR3 gene in human. However, it has been proposed by others, using evidence from the conserved syntenic position of several genes, that FGFR and FGF8/17/18 orthologues were linked in the ancestor of bilaterian animals [21]. Given the position of the FGFRL1 genes, close to FGFR orthologues and/or to FGF8/17/18 orthologues, we propose that FGFR, FGF8/17/18, and FGFRL1 genes were linked in Urbilateria. In *Nematostella *we did not find any orthologous genes of the bilaterian FGFR/FGFRL1/FGF8/17/18 syntenic block in the same scaffold as FGFRL1 with the exception of FGF8a and FGF8b. Nevertheless, the fact that these three genes are positioned close together in this species may suggest a functional link between them. It is noteworthy that the position of the *Drosophila *FGFRL1 putative gene confirms its orthology with the other species' FGFRL1, in spite of a poorly supported position in our phylogenetic tree reconstructions.

### FGFRL1 expression during amphioxus and mouse embryogenesis

To better understand the putative implication of FGFRL1 during embryonic development in chordates, we chose to analyze its expression pattern by whole mount *in situ *hybridization in two species: amphioxus, a cephalochordate placed at the base of the chordate clade; and mouse, a vertebrate for which only late embryonic expression has been described [22].

**Figure 5:**
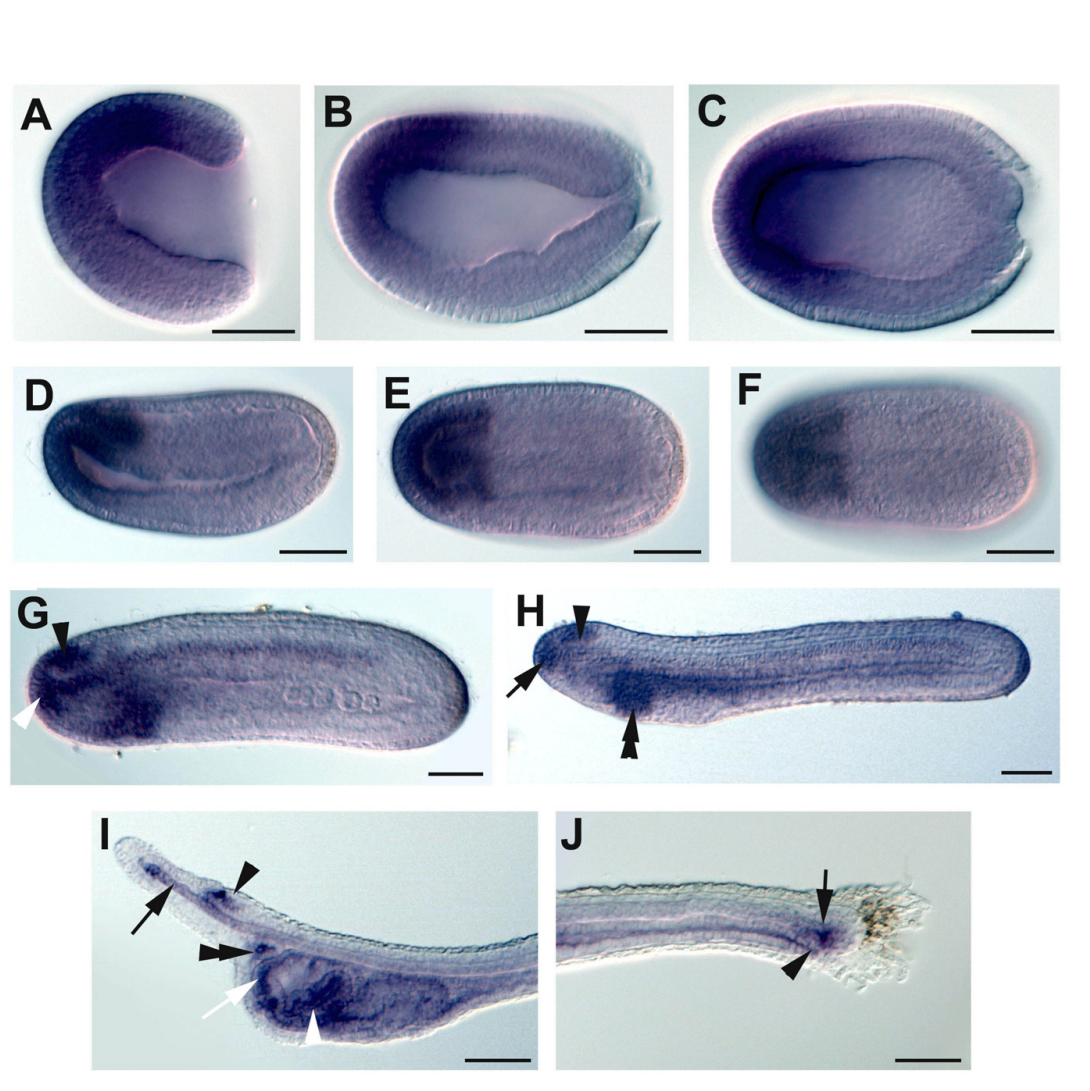
**Embryonic expression of amphioxus FGFRL1**. Anterior is towards the left, and dorsal is up in the side views. Scale bars = 50 μm. (A) Side view of a gastrula stage embryo showing high expression of FGFRL1 in the anterior dorsal region. (B) Side view of an early neurula stage embryo. (C) Dorsal view of the embryo shown in (B). (D) Side view of a mid-neurula stage embryo with expression in the anterior region of the mesendoderm and neuroectdoderm as well as in the most anterior epidermis. (E) and (D) Dorsal views of the embryo shown in (D). (G) Side view of a late neurula stage embryo with labelling in the notochord, mainly in its most anterior part (white arrowhead), in the cerebral vesicle (black arrowhead) as well as in the anterior-most epidermis and in the pharyngeal endoderm. (H) Side view of a late neurula stage embryo showing expression in the cerebral vesicle (black arrowhead), in the notochord, mainly in its most anterior region (black arrow), and in the endoderm, with a high level in the region of the club-shaped gland anlagen (double black arrowhead). (I) Side view of the head of a larva. FGFRL1 is expressed in the cerebral vesicle around the frontal eye (black arrowhead), in the notochord with a high level in the most rostral region (black arrow), in the pre-oral pit (double black arrowhead), in the anterior region of the pharyngeal cavity (white arrow) and in the club-shaped gland (white arrowhead). (J) Side view of the tail of the specimen shown in (I) with labelling in the tailbud (black arrow) and in the anus (black arrowhead).

In amphioxus, expression starts very early during embryogenesis since we can detect FGFRL1 mRNA from the 4 cell stage. Before gastrulation, expression is ubiquitous. In late gastrula a higher expression level is visible in the anterior dorsal part of the embryo (Figure 5A). In early neurula stage embryos, basal levels start to decrease and expression becomes concentrated in the anterior part of the embryo, with a more posterior limit in the dorsal region (Figure 5B-C). This pattern of expression is identical in mid-neurula stage embryos (Figure 5D-F), the posterior limit of the labelled region being sharper. In late neurula, expression becomes more restricted. Indeed, at this stage, labelling is detected in the cerebral vesicle, in the anterior epidermis, in the notochord, with a higher expression level in its anterior region and almost no expression in its most posterior part, and in the ventral and lateral pharyngeal endoderm (Figure 5G). At a later stage, just before mouth opening, the pattern is similar except that expression in the notochord is lower at the level of the trunk (Figure 5H). At this stage, expression in the endoderm is very high in the region that will give rise to the club-shaped gland, but absent in the region where the future gill slits will open. In the trunk endoderm, it expands towards the most posterior region. In the larva, expression is observed in the club-shaped gland, in the pre-oral pit, around the mouth and in the most anterior and dorsal part of the pharyngeal endoderm (Figure 5I). The notochord is also labelled in its most anterior part, as is part of the cerebral vesicle. In the posterior part of the larva there is expression in the endoderm, mainly in the anus, as well as in the tailbud (Figure 5J).

**Figure 6:**
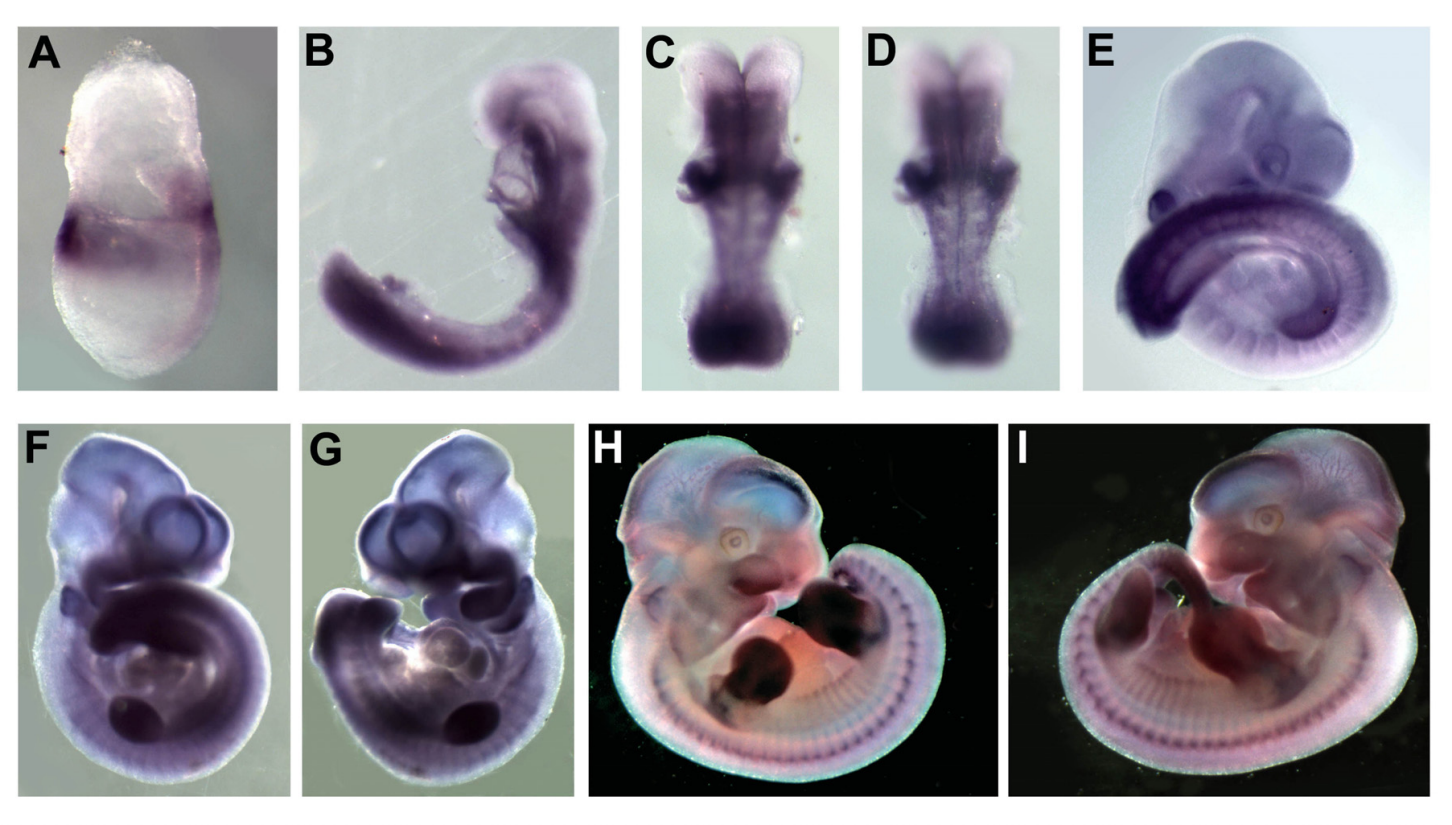
**Embryonic expression of mouse FGFRL1**. (A) E7.5 embryo showing FGFRL1 expression in the most anterior and posterior embryonic territories. (B) Side view of an E8.5 embryo. (C, D) Posterior view of the embryo shown in (B) with focus on the brain and the trunk respectively. At this stage expression is clearly observed in the somites, the tailbud and the anterior and posterior brain. (E) Side view of an E9.5 stage embryo showing expression of FGFRL1 in the frontonasal region, the otic vesicle, the somites, the forelimb buds and the tailbud. (F, G) Side views of an E10.5 stage embryo showing expression similar to E9.5 embryos. At this stage the hindlimb buds are developing and also express FGFRL1. (H, I) Side views of E11.5 embryos. Expression is detected in the frontal region of the brain, in the branchial arches, the otic vesicle, the tailbud and in the chondrogenic regions of the trunk and limbs.

In mouse, we have analyzed expression of FGFRL1 by whole mount *in situ *hybridization at embryonic days E7.5, E8.5, E9.5, E10.5 and E11.5. At the earliest stage, expression is restricted to the most anterior and posterior embryonic regions with a gradient from the axial to the lateral parts (Figure 6A). A sharp labelling of the anterior tip of endoderm is found, just below the rostral limit of the neural plate. At E8.5, expression is more widespread in both anterior and posterior regions. In the head, the foregut and otic placodes are heavily labelled, and less intense expression is found at the level of the rhombencephalon. In the posterior part of the embryo, expression is detected in the tailbud, the pre-somitic mesoderm and in dorsal-most part of the somites (Figure 6B-D). At E9.5 and E10.5, the frontonasal region of the brain, the branchial arches, the otic vesicle, the limb buds, the posterior part of the somites and the tailbud are labelled (Figure 6E-G). At E11.5, expression decreases in branchial arches as well as in the brain (Figure 6H-I). At this stage, expression is maintained in the otic vesicle as well as in the chondrogenic regions of the trunk and the limbs.

## Discussion

### FGFRL1 appeared early during metazoan evolution and was linked to genes of the FGF signalling pathway

First, our analysis of the planarian *nou-darake *sequence supported its *bona fide *orthology to the chordate FGFRL1 genes. Our subsequent search for other orthologues in complete genomes led us to identify FGFRL1 genes in many metazoan species. Although the position in the phylogenetic reconstructions is not well supported for thediploblastic sequences, the conserved structure of the protein and of the gene in *Nematostella vectensis*, and of the genomic position in insects, together with the reciprocal blast results, highly support the fact that these genes are FGFRL1 orthologues in these species. As we were unable to find FGFRL1 genes outside metazoans, even in the choanoflagellate *Monosiga brevicolis *genome, we conclude that FGFRL1 appeared early during metazoan evolution, and was present at least in the common ancestor of bilaterian and diploblastic animals, as is also the case for the FGF and FGFR gene families [2].

In our analysis of the genomic environment of FGFRL1 genes, we found that they are in the vicinity of FGF8/17/18 or FGFR, or both, in many genomes. It has previously been suggested that FGFR8/17/18 and FGFR orthologous genes were probably linked in the ancestor of bilaterian animals [1,21]. We propose that the FGFRL1 orthologue was also part of the same syntenic block in Urbilateria. In *Nematostella vectensis*, the *NvSprouty *gene is located between the FGFRL1 gene and the FGF8a gene (at position 1,09 Mb in the scaffold 51 represented in Figure 4) [2]. *Sprouty *homologues in vertebrates and *Drosophila *are known to be negative regulators of FGF signalling [23], and in *Nematostella*, expression of *NvSprouty *overlaps with that of several FGF ligands. It has therefore been proposed that the FGF-Sprouty feedback loop is conserved between cnidarian and bilaterian animals [2]. The fact that FGFRL1 is linked to FGF signalling genes in the genomes of cnidarians to bilaterians suggests that it may play a role in this pathway, although this still needs to be clearly demonstrated. Some functional data in the planarian *Dugesia japonica *indicates that this might be the case during regeneration in this species [14]. Indeed, expression data and RNAi experiments show that the FGFRL1 orthologue *nou-darake *is implicated in brain tissue induction during regeneration and that this induction is suppressed by double-strand RNA injection for FGFR1 and FGFR2. Moreover, overexpression of *nou-darake *in *Xenopus *embryos leads to developmental phenotypes that are very similar to those obtained after overexpression of XFD, a dominant-negative FGF receptor. Although the biochemical function of *nou-darake *still needs to be elucidated, it was subsequently proposed that it might be a modulator of FGF signalling [14].

### FGFRL1 embryonic expression sheds light on putative conserved and non-conserved functions in chordates

In vertebrates, the embryonic expression pattern of FGFRL1 has already been investigated in *Xenopus *[24] and in zebrafish, which possesses two genes, *fgfrl1a *and *fgfrl1b *[5,10]. In *Xenopus *as in mouse, FGFRL1 expression starts at the gastrula stage, whereas neither of the zebrafish duplicates is expressed before somitogenesis. However, in *Xenopus*, expression is restricted to the anterior region of the gastrula whereas we have found anterior and posterior expression in mouse. This disparity may reflect a difference in the early function of the FGFRL1 orthologues in these species, or may reflect the fact that the gastrulation process is divergent in mouse, zebrafish and *Xenopus*. During later stages, expression in the telencephalon, branchial arches, otic vesicle, and somites is shared among mouse and *Xenopus *FGFRL1 and zebrafish *fgfrl1a*. However, in zebrafish, expression in somites is transient whereas in mouse and *Xenopus*, expression starts early and continues into late development. In *Xenopus*, although expression is detected in the mid-hindbrain boundary and in the lens, as for zebrafish *fgfrl1a *and *fgfrl1b *respectively, this is not the case in mouse. While some expression domains appear conserved among the three vertebrates, the presence of clear differences suggests the existence of conserved and non-conserved functions during vertebrate development.

Interestingly, amphioxus and vertebrate FGFRL1 expression patterns share some similarities. Indeed, in the gastrula stage embryos of amphioxus, we observed high expression in the anterior part, similarly to what has been described in *Xenopus*. At later stages, expression in the cerebral vesicle and in parts of the pharyngeal endoderm in amphioxus can be considered as homologous to the expression in the anterior brain and in the branchial arches anlagen in *Xenopus*, zebrafish and mouse. However, the somites do not express FGFRL1 in amphioxus, a surprising result given that it is clearly detected at least transitorily in all vertebrates studied. It has also been proposed that FGFRL1 might be implicated in cartilage formation in vertebrates, and morpholino injection in zebrafish shows that both duplicates *fgfrl1a *and *fgfrl1b *play a role during pharyngeal arch development [10]. In vertebrates FGF3 and FGF8 are expressed in pharyngeal endoderm where they induce cartilage formation. Amphioxus does not have cartilage but, like FGFRL1, FGF8/17/18 is expressed in the pharyngeal endoderm [25], although overlap of their expression domains is not complete. On the other hand, as described recently in amphioxus, there is not a broad coexpression of cartilage marker genes in this region of the embryo [25]. These data might suggest that FGF8/17/18 and FGFRL1 coexpression in the pharyngeal endoderm, the first sign of a possible interaction between the two, was already present in the chordate ancestor, and that the two genes were recruited in vertebrates for cartilage formation in this region of the embryo.

### Conflicting functional data in vertebrates

Although the expression data we have obtained in mouse might indicate that FGFRL1 is implicated in cartilage formation, as is the case in zebrafish, the functional data that were available until very recently did not support this hypothesis. In zebrafish, morpholino injections for the two duplicates *fgfrl1a *and *fgfrl1b *show that they have a function in pharyngeal arch formation [10]. In contrast, knock-out (KO) of FGFRL1 in mouse, as first described by Baertschi and collaborators, caused animals to die early after birth due to respiratory failure associated with diaphragm alteration [11]. These animals do not show obvious skeletal abnormalities in spite of specific embryonic expression of FGFRL1 in cartilage anlagen. However, some data suggested that in human, as in zebrafish, FGFRL1 might be implicated in facial cartilage formation during embryogenesis. For example, two human patients having facial malformations caused by the deletion of the FGFRL1 gene, or by a frameshift mutation in its intracellular coding part, have been described [26,27]. These data, together with the results obtained in zebrafish, made the absence of skeletal abnormalities after FGFRL1 gene inactivation in mouse really puzzling. Finally, knock-out mutant mice, using a different construct, show craniofacial defects in addition to diaphragm malformations [12]. This strongly suggests that FGFRL1 implication in pharyngeal cartilages development is conserved in vertebrates, and that the first mutant described in mouse did not show a phenotype corresponding to full inactivation of this gene. Such a discrepancy could result from the inefficacy of the construct used to create the first FGFRL1 KO mutants, which only deletes exon 0 and exon 1, corresponding to the 5' UTR and to the signal peptide coding part of the mouse gene. Exons encoding the three Ig loops, as well as the transmembrane and cytoplasmic domains, are still present in mutant mice. One could imagine that an mRNA might be produced from an alternative 5' UTR, leading to partial protein production and function. This could explain the mild phenotype observed in these mutants compared to the more recently published ones.

## Conclusion

Our work shows for the first time that FGFRL1 has clear orthologues in many metazoan species, which is in stark opposition to previously published data [13,27]. Given the structural similarity of the FGFRL1 protein to FGFRs, and its genomic linkage with other members of the FGF signalling pathway from cnidarians to bilaterians, it is reasonable to propose that FGFRL1 is an actor of this pathway in metazoans, thus far neglected due to its supposed inexistence outside deuterostomes. The expression data in chordates support a conserved function in the pharyngeal endoderm, which has been shown in zebrafish by morpholino injections, and, more recently, by knock-out mutant constructs in mouse.

Data on the embryonic role of FGFRL1 orthologues outside the chordate group remain sparse. In the FlyBase database [28], we have found the expression pattern of the *Drosophila *FGFRL1 gene (named CG31431), but it is still unpublished. The expression pattern is very restricted throughout embryogenesis, which might indicate a specific function that still needs to be studied. With respect to *Caenorhabditis elegans*, we looked at the data available in WormBase [29] for the FGFRL1 orthologous gene (named Y102A11A.8). There is no embryonic expression description, and there is an absence of specific phenotype described for one RNAi experiment [30]. In planarians, no embryonic expression has been described, but functional data in *Dugesia japonica *strongly suggest interaction between FGFRL1 and FGF signalling during regeneration. Our work reveals the existence of a widely conserved gene, FGFRL1, whose characteristics may shed light on the most ancestral FGF functions. Detailed developmental studies are needed to decipher its full evolutionary history and the diversification of FGF signalling pathways in metazoans.

## Methods

### Genomic and databases sequences survey

We searched for FGFRL1 orthologous sequences using Blast with the mouse FGFRL1 peptide sequence and the planarian *nou-darake *sequence [31]. We first used BlastP on the predicted peptides and then TBlastN on the genomic sequences. We also used BlastP on Genbank sequences to look for more putative orthologues. The sequences obtained were then analyzed by BlastP to exclude false-positives. The genomic databases used are listed in Table 1.

### Phylogenetic analysis

Predicted amino acid sequences were aligned automatically using ClustalW [32] with manual correction in Seaview [33]. Phylogenetic reconstruction was done using amino acid alignments of the longest sequences found for each gene. Only complete sites (no gap, no X) were used. Phylogenetic trees were built using RaxML, under the JTT+I+G model, with 100 bootstrap repetitions on the Cipres Portal v1.15 [34].

### Isolation of FGFRL1 cDNA

Partial cDNAs from *B. lanceolatum *and mouse FGFRL1 were cloned by RT-PCR on adult RNA in pGEM-T Easy vector (Promega). An insert was then subcloned in pBluescript II SK (Stratagene) for subsequent RNA probe synthesis. Accession number for the *B. lanceolatum *sequence is FJ694966.

### Whole mount in situ hybridization

Ripe animals of the Mediterranean amphioxus (*B. lanceolatum*) were collected in Argelès-sur-Mer (France), and gametes were obtained by heat stimulation [35,36]. Fixation and whole-mount *in situ *hybridization were performed as described in [37], except the chromogenic reaction which was performed using BM Purple [38]. Mouse embryos were dissected in PBS, fixed in 4% paraformaldehyde (PFA) and whole mount *in situ *hybridization was performed according to [39].

## Authors' contributions

SB, IS, TL, JGF and HE conceived and designed the experiments and wrote the paper. SB, IS, and TL performed the experiments. SB, IS, TL, and HE analyzed the data. All authors read and approved the final manuscript.

## Supplementary Material

Additional file 1 **Phylogenetic analysis of the putative FGFRL1 orthologues**. Phylogenetic tree estimated under the JTT+I+G model (RAxML with rapid bootstrap analysis with 100 bootstrapping runs). All the FGFRL1 sequences described in the manuscript were included. VEGFR sequences from vertebrates were used as the outgroup. FGFR sequences are indicated in blue and FGFRL1 sequences in red. Sequences not included in Figure 2 are highlighted in yellow. The species abbreviations are as follows: *AEDAE: Aedes aegypti, ANOGA: Anopheles gambiae, BRABE: Branchiostoma belcheri, BRAFL: Branchiostoma floridae, BRUMA: Brugia malayi, CAEBR: Caenorhabditis briggsae, CAEEL: Caenorhabditis elegans, CAPSP: Capitella sp.I, CIOIN: Ciona intestinalis, CIOSA: Ciona savignyi, DANRE: Danio rerio, DAPPU: Daphnia pulex, DROME: Drosophila melanogaster, DROPS: Drosophila pseudoobscura, DUGJA: Dugesia japonica, GALGA: Gallus gallus, HOMSA: Homo sapiens, LOTGI: Lottia gigantea, MUSMU: Mus musculus, NEMVE: Nematostella vectensis, RATNO: Rattus norvegicus, STRPU: Strongylocentrotus purpuratus, TAKRU: Takifugu rubripes, TETNI: Tetraodon nigroviridis, TRICA: Tribolium castaneum, XENLA: Xenopus laevis, XENTR: Xenopus tropicalis*. Click here for file

Additional file 2 **Phylogenetic analysis of the putative FGFRL1 orthologues**. Phylogenetic tree estimated under the JTT+I+G model (RAxML with rapid bootstrap analysis with 100 bootstrapping runs). All the FGFRL1 sequences described in the manuscript were included, with the exception of FGFR orthologue sequences from protostomes. VEGFR sequences from vertebrates were used as the outgroup. FGFR sequences are indicated in blue and FGFRL1 sequences in red. Sequences not included in the Figure 2 are highlighted in yellow. The species abbreviations are as follows: *AEDAE: Aedes aegypti, ANOGA: Anopheles gambiae, BRABE: Branchiostoma belcheri, BRAFL: Branchiostoma floridae, BRUMA: Brugia malayi, CAEBR: Caenorhabditis briggsae, CAEEL: Caenorhabditis elegans, CAPSP: Capitella sp.I, CIOIN: Ciona intestinalis, CIOSA: Ciona savignyi, DANRE: Danio rerio, DAPPU: Daphnia pulex, DROME: Drosophila melanogaster, DROPS: Drosophila pseudoobscura, DUGJA: Dugesia japonica, GALGA: Gallus gallus, HOMSA: Homo sapiens, LOTGI: Lottia gigantea, MUSMU: Mus musculus, NEMVE: Nematostella vectensis, RATNO: Rattus norvegicus, STRPU: Strongylocentrotus purpuratus, TAKRU: Takifugu rubripes, TETNI: Tetraodon nigroviridis, TRICA: Tribolium castaneum, XENLA: Xenopus laevis, XENTR: Xenopus tropicalis*. Click here for file
