# Supplementary figures and images for: FGFRL1 is a neglected putative actor of the FGF signalling pathway present in all major metazoan phyla
Source: BMC Evol Biol. 2009 Sep 9;9:226. doi: 10.1186/1471-2148-9-226 (PMC2754479; doi:10.1186/1471-2148-9-226)

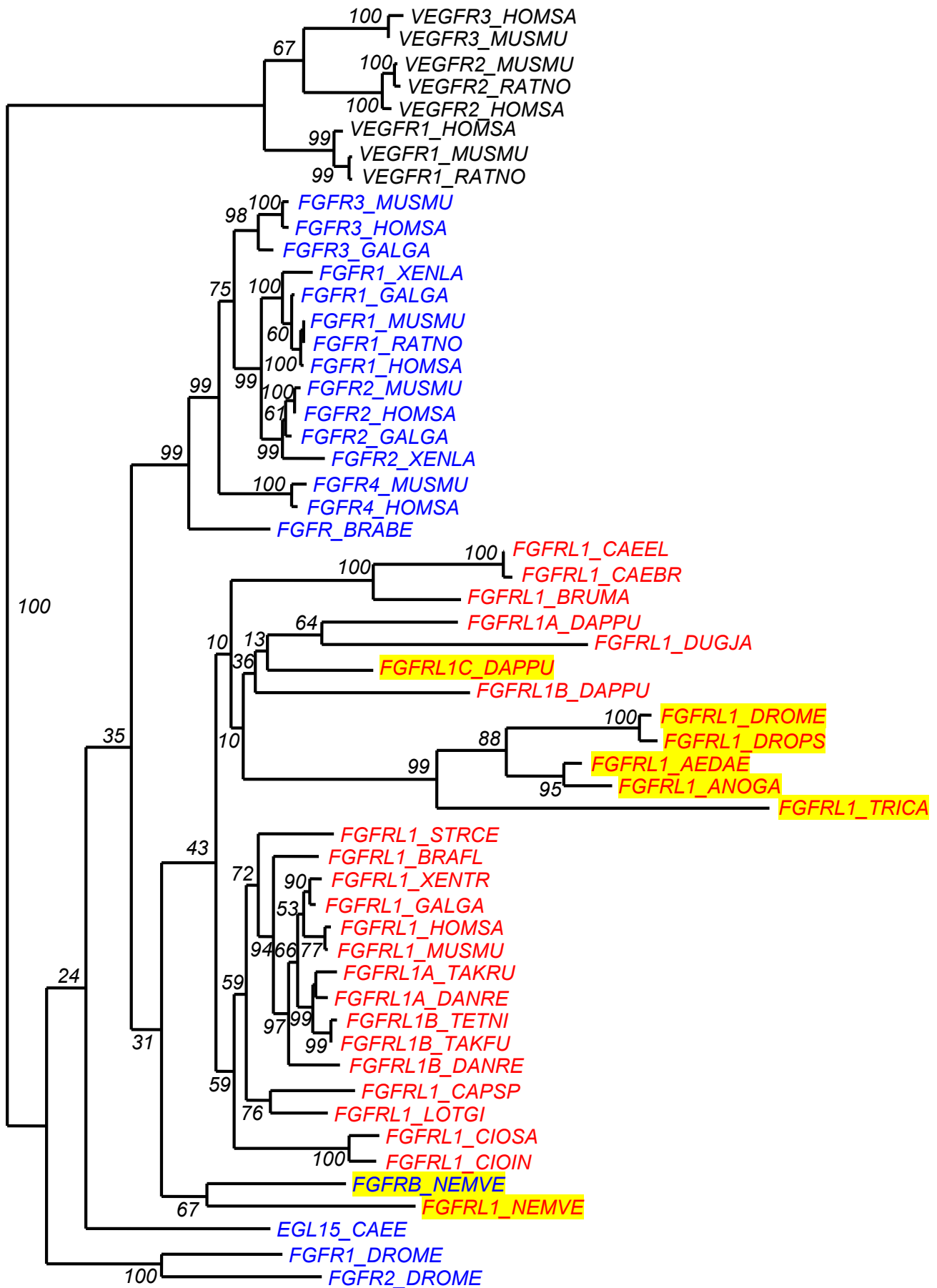

Supplement: Additional file 1 — Phylogenetic analysis of the putative FGFRL1 orthologues. Phylogenetic tree estimated under the JTT+I+G model (RAxML with rapid bootstrap analysis with 100 bootstrapping runs). All the FGFRL1 sequences described in the manuscript were included. VEGFR sequences from vertebrates were used as the outgroup. FGFR sequences are indicated in blue and FGFRL1 sequences in red. Sequences not included in Figure 2 are highlighted in yellow. The species abbreviations are as follows: AEDAE: Aedes aegypti, ANOGA: Anopheles gambiae, BRABE: Branchiostoma belcheri, BRAFL: Branchiostoma floridae, BRUMA: Brugia malayi, CAEBR: Caenorhabditis briggsae, CAEEL: Caenorhabditis elegans, CAPSP: Capitella sp.I, CIOIN: Ciona intestinalis, CIOSA: Ciona savignyi, DANRE: Danio rerio, DAPPU: Daphnia pulex, DROME: Drosophila melanogaster, DROPS: Drosophila pseudoobscura, DUGJA: Dugesia japonica, GALGA: Gallus gallus, HOMSA: Homo sapiens, LOTGI: Lottia gigantea, MUSMU: Mus musculus, NEMVE: Nematostella vectensis, RATNO: Rattus norvegicus, STRPU: Strongylocentrotus purpuratus, TAKRU: Takifugu rubripes, TETNI: Tetraodon nigroviridis, TRICA: Tribolium castaneum, XENLA: Xenopus laevis, XENTR: Xenopus tropicalis. [file 1471-2148-9-226-S1.pdf]

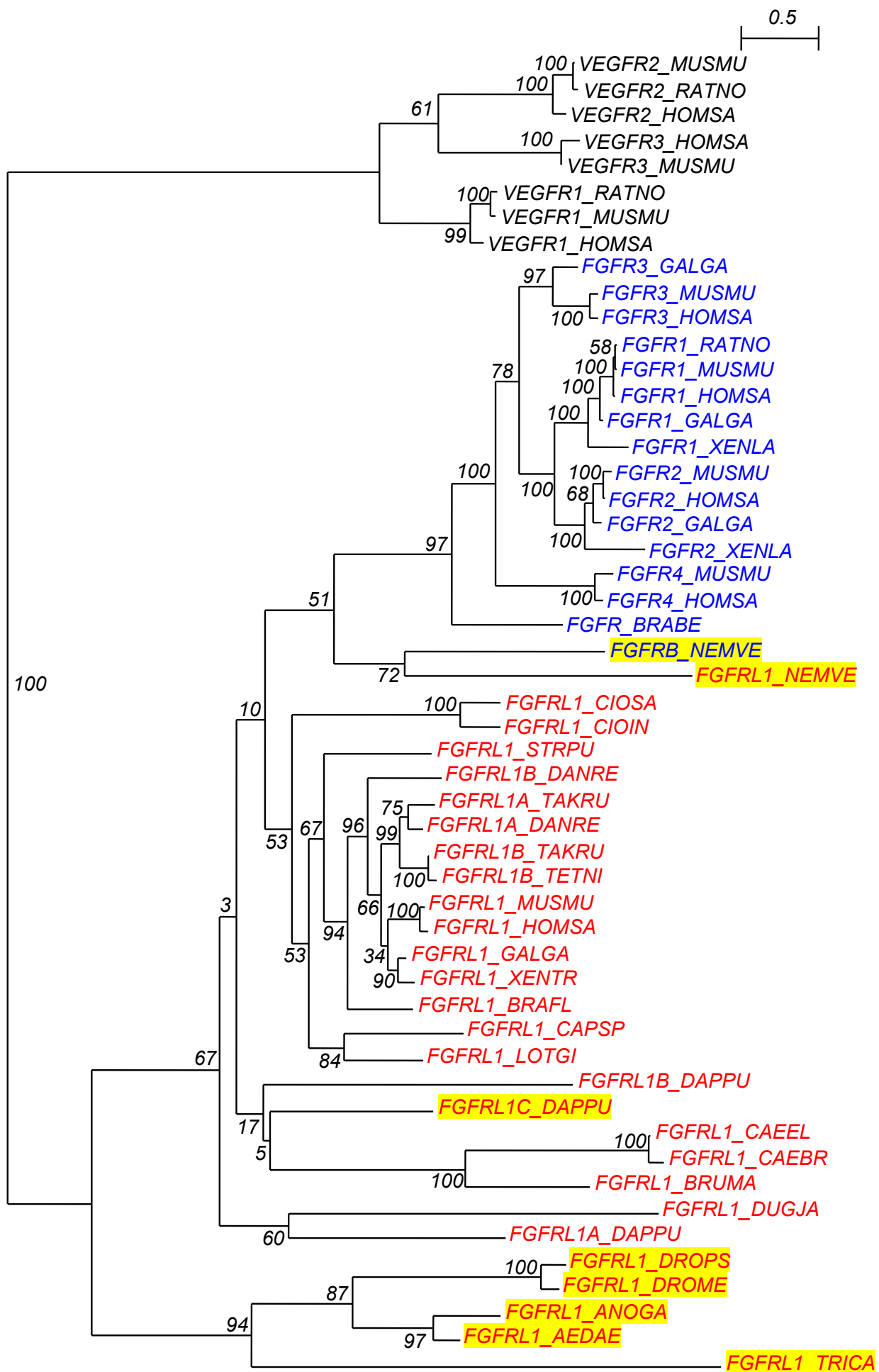

Supplement: Additional file 2 — Phylogenetic analysis of the putative FGFRL1 orthologues. Phylogenetic tree estimated under the JTT+I+G model (RAxML with rapid bootstrap analysis with 100 bootstrapping runs). All the FGFRL1 sequences described in the manuscript were included, with the exception of FGFR orthologue sequences from protostomes. VEGFR sequences from vertebrates were used as the outgroup. FGFR sequences are indicated in blue and FGFRL1 sequences in red. Sequences not included in the Figure 2 are highlighted in yellow. The species abbreviations are as follows: AEDAE: Aedes aegypti, ANOGA: Anopheles gambiae, BRABE: Branchiostoma belcheri, BRAFL: Branchiostoma floridae, BRUMA: Brugia malayi, CAEBR: Caenorhabditis briggsae, CAEEL: Caenorhabditis elegans, CAPSP: Capitella sp.I, CIOIN: Ciona intestinalis, CIOSA: Ciona savignyi, DANRE: Danio rerio, DAPPU: Daphnia pulex, DROME: Drosophila melanogaster, DROPS: Drosophila pseudoobscura, DUGJA: Dugesia japonica, GALGA: Gallus gallus, HOMSA: Homo sapiens, LOTGI: Lottia gigantea, MUSMU: Mus musculus, NEMVE: Nematostella vectensis, RATNO: Rattus norvegicus, STRPU: Strongylocentrotus purpuratus, TAKRU: Takifugu rubripes, TETNI: Tetraodon nigroviridis, TRICA: Tribolium castaneum, XENLA: Xenopus laevis, XENTR: Xenopus tropicalis. [file 1471-2148-9-226-S2.pdf]
